# Reliability of Force Plate Metrics During Standard Jump, Balance, and Plank Assessments in Military Personnel

**DOI:** 10.1093/milmed/usac387

**Published:** 2022-12-15

**Authors:** Chelsea Smith, Kenji Doma, Brian Heilbronn, Anthony Leicht

**Affiliations:** Royal Australian Army Medical Corps, Australian Army, Townsville, QLD 4811, Australia; College of Medicine and Dentistry, James Cook University, Townsville, QLD 4811, Australia; Sport and Exercise Science, James Cook University, Townsville, QLD 4811, Australia; Royal Australian Army Medical Corps, Australian Army, Townsville, QLD 4811, Australia; Sport and Exercise Science, James Cook University, Townsville, QLD 4811, Australia; Sport and Exercise Science, James Cook University, Townsville, QLD 4811, Australia; Australian Institute of Tropical Health and Medicine, James Cook University, Townsville, QLD 4811, Australia

## Abstract

**Introduction:**

Prevention of musculoskeletal injury is vital to the readiness, performance, and health of military personnel with the use of specialized systems (e.g., force plates) to assess risk and/or physical performance of interest. This study aimed to identify the reliability of one specialized system during standard assessments in military personnel.

**Methods:**

Sixty-two male and ten female Australian Army soldiers performed a two-leg countermovement jump (CMJ), one-leg CMJ, one-leg balance, and one-arm plank assessments using a Sparta Science force plate system across three testing sessions. Sparta Science (e.g., total Sparta, balance and plank scores, jump height, and injury risk) and biomechanical (e.g., average eccentric rate of contraction, average concentric force, and sway velocity) variables were recorded for all sessions. Mean ± SD, intraclass correlation coefficients (ICCs), coefficient of variation, and bias and limits of agreement were calculated for all variables.

**Results:**

Mean results were similar between sessions 2 and 3 (*P* > .05). The relative reliability for the Sparta Science (ICC = 0.28-0.91) and biomechanical variables (ICC = 0.03-0.85) was poor to excellent. The mean absolute reliability (coefficient of variation) for Sparta Science variables was similar to or lower than that of the biomechanical variables during the CMJ (1-10% vs. 3-7%), one-leg balance (4-6% vs. 9-14%), and one-arm plank (5-7% vs. 12-17%) assessments. The mean bias for most variables was small (<5% of the mean), while the limits of agreement varied with most unacceptable (±6-87% of the mean).

**Conclusions:**

The reliability of most Sparta Science and biomechanical variables during standard assessments was moderate to good. The typical variability in metrics documented will assist practitioners with the use of emerging technology to monitor and assess injury risk and/or training interventions in military personnel.

## INTRODUCTION

The prevention of musculoskeletal injury (MSKI) is pivotal to the improvement of the readiness, performance, and long-term health of military personnel.^1,2^ Forty percent of clinical presentations are attributed to preventable MSKI in the Australian Defence Force^3^ and account for nearly 60% of soldier’s limited duty days in the U.S. Army,^4^ costing billions of dollars and compromising the readiness and occupational performance of military personnel.^1^ Factors that place military personnel at risk of MSKI include poor aerobic fitness, balance, muscular endurance, power, and strength.^2,5^ Enhancement of these factors contributes to soldier’s performance of numerous physically demanding tasks, including carrying heavy loads over long distances and uneven terrain to sprinting across the battlefield to seek cover and negotiate obstacles,^2,6,7^ while concurrently reducing the risk of MSKI.^2,5^

Prescreening assessments are essential to identify MSKI risk factors and/or physical performance, which inform the development and implementation of appropriate injury prevention programs.^5^ Currently, the physical performance of military personnel in multiple countries, including Australia and the USA, is assessed via running and generic activities (e.g., push-ups, sit-ups, timed run, etc.) during regular physical fitness tests.^7^ Several studies have reported an association between poor performance on these physical tests (e.g., timed runs) and an increased MSKI risk.^8–10^ Although these tests primarily focus on the fitness domains of aerobic and muscular endurance, they may not address other fitness domains, such as balance, muscular power, and strength, aspects crucial for reducing MSKI risk.^7^ Subsequently, the use of specialized systems in the assessment of strength, power, balance, and functional movement with MSKI risk has been investigated in the military.^11–15^ However, only moderate associations, with low sensitivity and specificity, between these tests and injury risk in soldiers have been found.^11–15^ Despite these moderate associations, the use of these systems with elite athletes,^11^ and the persisting high injury burden within the military,^3,4^ interest in such systems persists.^16–18^

One new system is a commercial, off-the-shelf force plate (Sparta Science Inc., Menlo Park, CA, USA) that assesses countermovement jump (CMJ), single-leg balance, and one-arm plank performances that ascribe injury risk based on predictive algorithms.^16,17,19^ The Sparta Science software draws upon an ever-growing database of jump, balance, and plank performances. The Sparta Science system focuses on the assessment of lower limb muscular power, balance, and core stability, fitness domains significantly associated with MSKI risk.^5^

Previous research on the Sparta Science system in high school, college, and professional athletes identified that poor performance on the CMJ, balance, and core stability/plank assessments was associated with a greater risk of lower extremity injury,^19^ concussions,^20^ and trunk/spinal injury, respectively.^21^ This prognostic ability was based upon the reliability of the Sparta Science system within athletic populations that who reported moderate-to-good intraclass correlation coefficient (ICC) values (0.61-0.90) and good-to-poor coefficient of variation (CV) values (2.7-21.3%) for several biomechanical variables.^22–25^

Recently, the use of this system was extended to an occupational setting (i.e., special warfare trainees) where the reliability of some unique Sparta Science scores and CMJ subdomain values was reported.^26^ Despite this novel focus, this study examined a small sample size (*n* = 12) with no examination of balance and core stability, important fitness domains significantly associated with MSKI risk.^5,26^ Given the system’s portability, which is well suited for a mobile military force, identification of the reliability or normal biological/measurement variation of the Sparta Science system’s range of variables would aid the military to identify practically meaningful changes during injury risk monitoring and future interventions.^22,27^ Subsequently, the aim of this study was to identify the reliability of the unique and standard assessments (i.e., CMJ, balance, and core stability) using the Sparta Science system for Australian Army personnel. We hypothesized that the reliability of all variables would be good (i.e., CV > 5%, ICC > 0.75, mean bias <5% of mean, and limits of agreement [LOAs] <5% of mean) in Australian Army personnel.

## METHODS

### Experimental Approach to the Problem

This study employed a repeated measures design to determine intersession reliability of two-leg CMJ, one-leg CMJ, one-leg balance, and one-arm plank assessments on a force plate. Participants completed three testing sessions with each separated by at least 48 h (range 2-11 days apart). All participants attended the testing location wearing normal physical training or operational attire and reported their level of muscle soreness during completion of a body weight squat using a 1-10 scale (i.e., 1 = “not sore”; 10 = “very, very sore”).^28^ At the start of each testing session, participants performed a standardized warm-up consisting of dynamic stretches, mobility exercises, and warm-up jumps, under the guidance of qualified exercise scientists and army physical training instructors.

### Participants

Sixty-two male and 10 female, full-time, Australian Army soldiers (age: 24.8 ± 5.7 years, height: 1.78 ± 0.09 m, mass: 79.7 ± 11.7 kg) volunteered to participate in this study. A sample size greater than 68 was sought to achieve a minimum ICC of 0.7.^29^ Participants were from a variety of combat operational units, were fully qualified within their individual employment categories, were of a deployable medical employment classification, and were free of injury as assessed by a prescreening health questionnaire. Leg and arm domination was identified as the limb that participants used for kicking and writing/throwing, respectively. Before testing, all participants were provided with written and verbal information about the study and provided written informed consent.

### Procedures

All assessments were performed by participants without shoes (i.e., barefoot) and using a commercially available piezoelectric force plate with a sampling frequency of 1,000 Hz (Bertec/Sparta, SSFP01; Sparta Science, Menlo Park, CA, USA). All data were collected and processed using commercially available software (Sparta Home 2.0, Sparta Science, Menlo Park, CA, USA). The force plate was self-calibrated according to the manufacturer’s specifications before testing and was zeroed before data collection.

#### CMJ assessments

After the standardized warm-up, participants completed four, maximal effort two-leg CMJ on the force plate. Each participant was instructed to stand on the force plate before waiting for an auditory cue, indicating stabilization of body weight, at which time the participant performed a CMJ with one two-arm swing (Supplementary Fig. S1). Ten-second rest intervals were provided between successive jumps. The greatest total Sparta score, load score, explode score, drive score, jump height (cm), injury risk score, average eccentric rate of contraction (AERC, N/s), average concentric force (ACF, N/kg), and concentric vertical impulse (CVI, Ns/kg) were obtained from the CMJ.^16–18^ The total Sparta, load, explode, and drive scores represented population-normalized *T*-scores generated using the Sparta Home 2.0 software, with the latter three scores derived from the AERC, ACF, and CVI results, respectively.^16,17,19^ The population-normalized *T*-scores were incorporated into a predictive algorithm to determine the participant’s injury risk score.^16,17,19^ The procedure was repeated for participant’s dominant and nondominant (ND) legs separately during one-leg CMJ as the last assessment of the session (Supplementary Fig. S2).

#### Balance and plank assessments

Following the two-leg CMJ assessments, participants’ balance and core stability were assessed on the force plate using one-leg balance and one-arm plank tests, respectively. For the balance tests, each participant stood on the force plate with 2 feet and eyes closed to establish a baseline reading. An auditory cue then indicated to the participant to balance on their right leg (lifting the left leg) for 20 s (Supplementary Fig. S3). A second auditory cue signaled the end of the 20-s trial. The participant then stepped off the force plate to reset. The procedure was repeated on the left leg. In total, four trials (two per limb) were completed. The participant’s total Sparta score and lowest/best sway velocity (m/s) were recorded for dominant and ND legs.^24^ The ratio of the participants’ best dominant to ND sway velocity was also calculated. In accordance with the manufacturer’s protocols, participants were allowed to re-establish their elevated foot with the ground, if needed briefly, to maintain balance during the assessment trial. Participants were encouraged to minimize this re-establishment activity during the balance trials.

For core stability, participants performed a one-arm plank test on the force plate. Participants stepped onto the force plate with both feet and stood still for the assessment of body mass. When prompted, the participant repositioned into a push-up/plank position with both hands on the plate and hands and feet shoulder width apart. Upon hearing an auditory cue, participants balanced on their right hand (picking up the left arm) for 20 s (Supplementary Fig. S4). A second auditory cue signaled the end of the 20-s trial. The participant removed both hands from the force plate to reset. The procedure was repeated with the left arm and so forth until four trials (two per limb) were completed. The participants’ total Sparta score and best and average sway velocities (m/s) for dominant and ND arms, as well as the ratio of their best dominant to ND sway velocity, were obtained.

### Statistical Analyses

Data are presented as mean ± SD, unless otherwise stated, with the normality of data confirmed using the Shapiro–Wilk test.^30^ Differences between sessions were determined via one-way repeated measures ANOVA and Bonferroni *post hoc* tests (Jamovi, version 2.3, Sydney, Australia). To determine the magnitude of difference between sessions, the effect size (ES) was calculated using Cohen’s *d* (Jamovi, version 2.3) and values were interpreted as follows: <0.20 (trivial), 0.20-0.59 (small), 0.60-1.19 (moderate), 1.20-1.99 (large), 2.00-4.00 (very large), and >4.00 (extremely large).^31^ Relative or intersession reliability was determined via ICCs (ICC_3,1_; single-rating, absolute-agreement; two-way mixed-effects model) calculated using SPSS (IBM, version 27, IL, USA). The level of reliability was evaluated based upon the 95% CIs of the ICC estimate using the following thresholds: excellent (>0.90), good to excellent (0.75-1.00), good (0.75-0.90), moderate to good (0.50-0.90), moderate (0.50-0.75), poor to moderate (0.00-0.75), and poor (<0.50).^32^ Absolute reliability or measurement error was established using the CV with relevant 95% CI, and values <5%, 5-10%, and >10% were considered good, moderate, and poor, respectively.^33^ The level of agreement between sessions was determined via group bias and LOAs,^34^ with the LOAs equivalent to the minimum detectable change.^25,35^ The bias ± LOAs were analyzed to assist practitioners to identify meaningful differences with interventions when utilizing the Sparta Science system and these standard assessments. For the purpose of this study, a mean bias of <5% of the mean and an LOA value of ± 5% of the mean (i.e., <10% total range) were interpreted as acceptable agreement. The level of significance for all analyses was set at *P *≤ .05.

## RESULTS

### Intersession Comparisons

Before assessments, mean muscle soreness was rated as low on the 10-point delayed onset muscle soreness (DOMS) scale with significantly greater soreness during sessions 2 (3.5 ± 1.9 vs. 2.6 ± 1.5; small ES = −0.51; 95% CI [−0.75, −0.26]) and 3 (3.9 ± 2.1 vs. 2.6 ± 1.5; trivial ES = −0.16; 95% CI [−0.39, 0.07]) compared to session 1. The mean results of Sparta Science (e.g., total Sparta score, load, etc.; Table I) and biomechanical variables (e.g., AERC, ACF, etc.; Table II) were similar between sessions 1 and 2 (*P* > .05) except for two-leg CMJ injury risk score (trivial ES = 0.27, 95% CI [0.03-0.51]). Likewise, the results for Sparta Science (Table I) and biomechanical variables (Table II) were similar between sessions 2 and 3 and sessions 1 and 3 (*P* > .05).

**Table I.** Mean and Coefficient of Variation Results for the Unique Sparta Science Assessments Conducted Three Times Over 2-14 Days

	Mean (SD) (Sess. 1)	Mean (SD) (Sess. 2)	Mean (SD) (Sess. 3)	CV (95% CI) (Sess. 1 vs. 2)	CV (95% CI) (Sess. 2 vs. 3)	CV (95% CI) (Sess. 1 vs. 3)
Two-leg CMJ (*n* = 70-72)						
Sparta score	77.6 (5.2)	78.0 (4.8)	78.4 (4.6)	2.25 (1.81-2.69)	2.01 (1.61-2.41)	2.81 (2.27-3.36)
Load score	44.2 (7.2)	43.4 (6.5)	42.4 (6.0)	6.23 (4.74-7.71)	5.73 (4.39-7.08)	7.42 (5.61-9.24)
Explode score	45.2 (10.6)	45.0 (9.1)	44.0 (7.9)	6.78 (5.07-8.50)	6.18 (4.98-7.38)	8.75 (6.57-10.92)
Drive score	43.7 (13.2)	44.3 (12.4)	45.0 (11.4)	9.69 (7.77-11.60)	11.07 (8.60-13.55)	13.35 (10.50-16.20)
Jump height (cm)	37.3 (7.6)	37.5 (7.8)	37.3 (7.6)	5.32 (4.01-6.64)	4.73 (3.88-5.57)	6.62 (5.18-8.07)
Injury risk score	1.7 (1.2)	1.6 (1.0)	1.4 (1.0)*	26.94 (16.67-37.21)	32.24 (21.77-42.70)	34.84 (23.83-45.86)
One-leg—dominant (*n* = 68-70)						
Sparta score	67.1 (3.7)	67.6 (3.5)	67.9 (3.3)	1.83 (1.43-2.23)	2.12 (1.62-2.63)	2.28 (1.66-2.90)
Load score	35.4 (4.1)	35.4 (3.9)	34.9 (3.4)	5.16 (4.05-6.27)	4.47 (3.73-5.22)	5.29 (4.13-6.45)
Explode score	24.1 (6.3)	24.0 (5.9)	23.7 (5.6)	8.04 (6.37-9.70)	7.33 (5.88-8.77)	9.914 (7.89-11.93)
Drive score	38.0 (16.5)	40.1 (13.9)	40.8 (13.2)	18.50 (13.90-23.08)	15.29 (11.70-18.89)	22.34 (17.26-27.42)
Jump height (cm)	17.5 (4.0)	18.2 (3.8)	18.0 (3.7)	6.95 (5.56-8.34)	6.43 (5.26-7.61)	8.24 (6.92-9.57)
Injury risk score	2.3 (1.5)	2.2 (1.3)	2.1 (1.4)	25.79 (18.24-33.34)	23.74 (15.90-31.58)	29.38 (21.10-37.65)
One-leg—ND (*n* = 66-69)						
Sparta score	66.9 (4.0)	67.4 (3.7)	67.9 (3.6)	2.17 (1.64-2.70)	2.30 (1.72-2.88)	2.70 (2.01-3.39)
Load score	35.3 (3.8)	35.0 (3.3)	35.0 (3.3)	5.25 (4.11-6.39)	4.25 (3.28-5.21)	5.38 (4.21-6.56)
Explode score	23.6 (6.6)	23.7 (6.1)	23.8 (6.0)	9.92 (7.10-12.75)	7.89 (6.32-9.46)	10.72 (8.09-13.34)
Drive score	39.0 (16.1)	40.1 (14.5)	41.6 (14.4)	19.69 (15.21-24.17)	16.00 (11.83-20.16)	21.98 (16.67-27.29)
Jump height (cm)	17.8 (4.4)	17.9 (4.0)	18.1 (3.8)	7.36 (5.89-8.82)	6.85 (5.49-8.20)	9.64 (8.03-11.26)
Injury risk score	2.3 (1.5)	2.2 (1.5)	2.2 (1.6)	23.39 (15.80-30.99)	18.28 (11.34-25.21)	26.86 (18.70-35.03)
Balance (*n* = 72)						
Dominant leg score	48.6 (6.4)	48.7 (5.7)	49.2 (5.8)	5.43 (4.16-6.71)	4.25 (3.24-5.25)	5.93 (4.67-7.19)
ND leg score	49.8 (6.2)	49.6 (6.3)	50.1 (6.1)	5.39 (4.12-6.66)	4.91 (3.79-6.02)	6.08 (5.03-7.14)
Plank (*n* = 66-68)						
Dominant arm score	46.7 (6.7)	47.9 (6.3)	48.2 (6.9)	6.91 (5.19-8.62)	6.46 (4.82-8.10)	7.04 (5.46-8.62)
ND arm score	48.0 (5.5)	48.0 (6.1)	48.0 (5.4)	5.49 (4.08-6.90)	5.51 (4.42-6.60)	5.89 (4.70-7.08)

Abbreviations: cm: centimeters; CV: coefficient of variation; *n*: number of participants; SD: standard deviation; Sess.: session; *P < .05 vs. sess. 1.

**Table II.** Mean and Coefficient of Variation Results for Sparta Science Biomechanical Assessments Conducted Three Times Over 2-14 Days

	Mean (SD) (Sess. 1)	Mean (SD) (Sess. 2)	Mean (SD) (Sess. 3)	CV (95% CI) (Sess. 1 vs. 2)	CV (95% CI) (Sess. 2 vs. 3)	CV (95% CI) (Sess. 1 vs. 3)
Two-leg CMJ (*n* = 61-64)						
AERC peak (N/s)	4,710 (1,884)	4,422 (1,779)	4,124 (1,602)	15.73 (12.77-18.68)	18.85 (14.05-23.65)	20.90 (16.00-25.80)
ACF peak (N/kg)	19.3 (1.9)	19.2 (1.8)	19.1 (1.6)	2.93 (2.40-3.45)	3.07 (2.47-3.67)	3.92 (3.19-4.64)
CVI peak (Ns/kg)	5.9 (0.5)	5.9 (0.5)	6.0 (0.5)	3.02 (2.40-3.64)	3.87 (3.03-4.71)	4.85 (3.88-5.82)
One-leg—D (*n* = 60-63)						
AERC peak (N/s)	2,455 (1,140)	2,279 (936)	2,276 (897)	23.57 (18.88-28.26)	18.36 (15.04-21.69)	24.36 (19.81-28.90)
ACF peak (N/kg)	15.5 (1.4)	15.3 (1.2)	15.2 (1.1)	2.89 (2.31-3.47)	2.64 (1.91-3.38)	3.64 (2.81-4.46)
CVI peak (Ns/kg)	5.8 (0.8)	5.9 (0.7)	5.9 (0.7)	5.57 (4.37-6.77)	4.47 (3.43-5.51)	6.69 (5.27-8.10)
One-leg—ND (*n* = 59-63)						
AERC peak (N/s)	2,429 (1,202)	2,169 (892)	2,224 (963)	22.41 (17.32-27.50)	19.19 (14.97-23.40)	22.52 (18.46-26.60)
ACF peak (N/kg)	15.2 (1.3)	15.2 (1.1)	15.3 (1.2)	2.77 (2.15-3.40)	2.34 (1.77-2.91)	3.11 (2.56-3.66)
CVI peak (Ns/kg)	5.9 (0.8)	5.9 (0.6)	5.9 (0.7)	5.46 (4.08-6.85)	5.19 (3.93-6.45)	6.68 (5.12-8.23)
Balance (*n* = 63-65)						
Sway velocity best—D (m/s)	0.10 (0.03)	0.10 (0.02)	0.10 (0.02)	11.56 (9.55-13.58)	9.04 (7.31-10.77)	12.74 (10.07-15.41)
Sway velocity best—ND (m/s)	0.10 (0.03)	0.10 (0.03)	0.10 (0.03)	11.94 (9.25-14.63)	10.01 (8.29-11.73)	14.25 (11.70-16.79)
D:ND ratio sway velocity best	1.06 (0.19)	1.04 (0.19)	1.05 (0.16)	11.01 (8.26-13.75)	11.96 (9.51-14.38)	13.01 (10.31-15.71)
Plank (*n* = 59-63)						
Sway velocity best—D (m/s)	0.03 (0.01)	0.03 (0.01)	0.03 (0.01)	15.77 (11.66-19.88)	14.42 (10.33-18.51)	16.56 (11.92-21.21)
Sway velocity best—ND (m/s)	0.03 (0.01)	0.03 (0.01)	0.03 (0.01)	14.26 (10.68-17.83)	14.61 (11.19-18.02)	15.31 (11.42-19.22)
D:ND ratio sway velocity best	1.04 (0.25)	0.99 (0.18)	1.00 (0.20)	13.62 (10.21-17.03)	11.99 (9.51-14.46)	14.16 (10.28-18.04)

Abbreviations: ACF: average concentric force; AERC: average eccentric rate of contraction; CV: coefficient of variation; CVI: concentric vertical impulse; D: dominant; *n*: number of participants; m/s: meters per second; N/kg: Newton per kilogram; N/s: Newton per second; ND: nondominant; Ns/kg: Newton second per kilogram; SD: standard deviation; Sess.: session.

### Absolute Reliability

The mean absolute reliability (CV) for the Sparta Science variables during the two-leg and one-leg CMJ ranged from 1 to 10% (i.e., moderate-good) across all sessions, except for injury risk score (18-35%) and drive score (10-22%) (Table I), which were poor. For the biomechanical variables, the CV was 3-7% (i.e., moderate-good) across all sessions, except for AERC (16-24%) (Table II), which was poor. For the balance assessments, the mean CV was poor to good with the Sparta Science variables lower (4-6%, Table I) compared to the biomechanical variables (9-14%, Table II), indicating better absolute reliability. Similarly, for the plank assessments, the mean CV was poor to moderate with lower CV observed for the Sparta Science variables (5-7%, Table I) compared to the biomechanical variables (12-17%, Table II).

The mean absolute reliability (CV) for the assessment of dominant and ND legs/arms was similar during each of the balance and plank assessments (Table I and II). Generally, the lowest CV for all variables was evident during the session 2 vs. 3 comparisons, whereas the greatest CV occurred during the session 1 vs. 3 comparisons (Table I and II).

### Relative Reliability

The relative reliability for the Sparta Science (ICC = 0.28-0.91, Table III) and biomechanical (ICC = 0.03-0.85, Table IV) variables ranged from poor to excellent. In particular, the CMJ assessments (e.g., two-leg drive, two-leg jump height, and one-leg dominant jump height) displayed the greatest reliability (i.e., good-to-excellent ICCs) compared to the other variables. Overall, the ICCs for the session 1 vs. 3 comparisons of most variables were lower when evaluated against the session 1 vs. 2 and session 2 vs. 3 contrasts.

**TABLE III.** Intraclass Correlation and Bias ± Limit of Agreement Results for the Unique Sparta Science Assessments Conducted Three Times over 2–14 Days

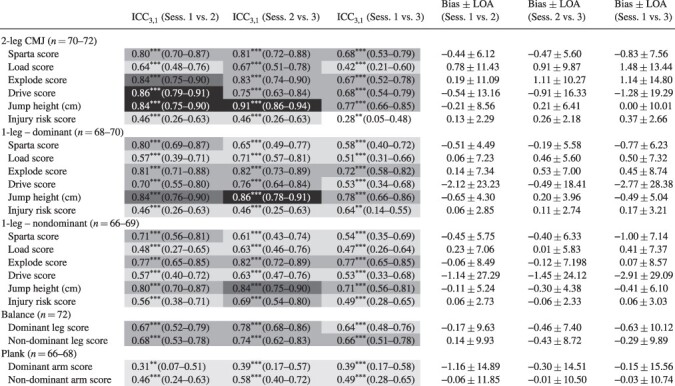

Abbreviations: Sess. = session, LOA = limit of agreement.

Values are mean (95% confidence intervals); *n* = number of participants; ** *P*<.01, *** *P*<.001.

ICC_3,1_ – intraclass correlation (single-rating, absolute-agreement, 2-way mixed-effects model) with the level of reliability was evaluated using the following general guideline based upon the 95% confidence interval of the ICC estimate.

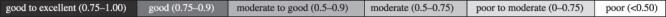

**TABLE IV.** Intraclass Correlation and Bias ± Limit of Agreement Results for Sparta Science Biomechanical Assessments Conducted Three Times over 2–14 Days

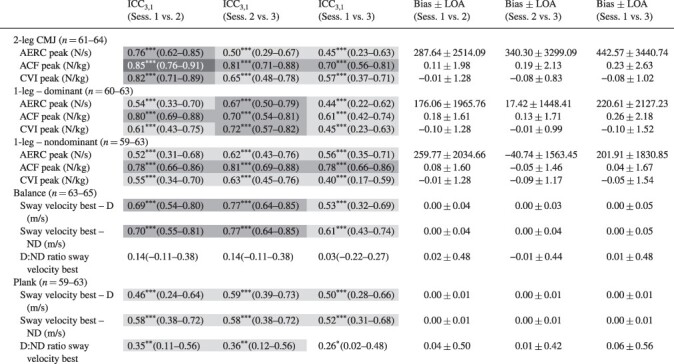

Abbreviations: Sess. = session, LOA = limit of agreement, AERC = average eccentric rate of contraction, N = Newton per second, ACF = average concentric force, N/kg = Newton per kg, CVI = concentric vertical impulse, N/kg = Newton second per kilogram, D = dominant, m/s = meters per second, ND = nondominant.

Values are mean (95% confidence intervals); *n* = number of participants; * *P*<.05, ** *P*<.01, *** *P*<.001.

ICC_3,1_ – intraclass correlation (single-rating, absolute-agreement, 2-way mixed-effects model) with the level of reliability was evaluated using the following general guideline based upon the 95% confidence interval of the ICC estimate.

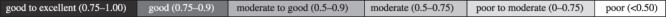

### Level of Agreement

The mean bias for the Sparta Science (Table III) and biomechanical variables (Table IV) was small (<5% of the mean) with the exception of injury risk score, one-leg CMJ drive score, and AERC (i.e., ∼5-20% of the mean). The LOAs for all variables were unacceptable (∼±6-87% of the mean, Table III and IV) and indicated that large changes in these variables were needed to indicate meaningful adjustments for future interventions. In addition, the mean bias ± LOA results for the session 1 vs. 3 comparisons were larger than the session 1 vs. 2 and sessions 2 vs. 3 contrasts.

## DISCUSSION

The aim of this study was to identify the intersession reliability of the Sparta Science and biomechanical variables for the CMJ, one-leg balance, and one-arm plank assessments in Australian Army personnel. Despite similar mean values for most variables across three repeat sessions, varying levels of reliability were noted for each assessment, thus partially supporting our hypothesis. Notably, greater absolute reliability was evident for the Sparta Science compared to the biomechanical variables (except the drive and injury risk scores). Relative reliability measures were poor to moderate for most variables, whereas the mean bias was small and the LOA results were unacceptable. The current results highlight the variability present for the Sparta Science and biomechanical parameters within a military population and will assist practitioners to detect meaningful differences using this technology in future interventions.

To date, most studies examining the reliability of the Sparta Science system have reported good reliability during CMJ, one-leg balance, and one-arm plank assessments,^22–26^ indicating the potential of the system to assess MSKI risk. However, these studies have focused on an elite athletic, homogenous population who are highly familiar with the movement patterns executed during the Sparta Science assessments and/or have superior abilities within these associated fitness domains.^22–25^ For example, Teske et al.^25^ reported good reliability of the Sparta Science metrics during the CMJ assessment (ICC = 0.78-0.83) for professional baseball players who likely perform similar movement patterns to the CMJ as part of their sprint training (e.g., plyometrics).^25^ Conversely, these movement patterns are not consistently part of military physical training that predominantly involves a traditional aerobic/endurance focus.^2^ Such discrepancies between regular training modalities and assessments may result in inconsistent reliability for such assessments in military personnel.^2^ Nonetheless, excellent (ICC > 0.97) reliability of the Sparta Science variables during the CMJ assessment in a small number of special warfare trainees (i.e., homogenous group) was reported recently.^26^ This level of reliability was comparable to that of elite athletes despite potential dissimilar movement patterns between military and elite athlete populations.^25^ Therefore, participant experience with the CMJ movement patterns may only partially contribute to the reliability of the Sparta Science metrics with other factors such as homogeneity of the population, requiring further examination. Despite the potential influences of these other factors, the Sparta Science variables in the current study exhibited moderate-to-good reliability (ICC 0.42-0.91), with the greatest reliability for two-leg CMJ total Sparta and explode scores (ICC >0.80). These reliability results along with those of others^26^ support the utility of the Sparta Science system to assess neuromuscular performance indices associated with MSKI risk in a military population.^26^

Although some of the specific CMJ results were consistent in the current study, the drive and injury risk scores were the least reliable and potentially impacted by the CMJ movement complexity.^16,18,36^ The drive variable during the take-off phase, which represents the CVI, includes both the concentric force of the jump and the duration of the transition period with variations in either or both components inflating any unreliability issues.^16,18,36^ In contrast, the load and explode variables include simple actions during the CMJ, with minimal variations in these likely resulting in greater reliability in comparison to the drive metric.^16,18,36^ Regardless, the drive variable is an important indicator of hip/thoracic mobility with poor mobility being a risk factor for groin, calf, and hamstring strains.^18^ Consequently, consideration of the drive variable when using the Sparta system to assess MSKI risk may still be practical for practitioners, as long as the lower reliability of this variable is acknowledged. Finally, given that the Sparta-derived injury risk score represented a proprietary defined amalgamation of scores, including the drive score, it was not surprising to observe the inconsistent reliability of this metric. Further development of this metric may improve its applicability as an assessment tool for performance and MSKI risk in military personnel.

Although the reliability of most Sparta Science variables during the two-leg CMJ was good, some similarities were observed for the one-leg CMJ Sparta Science variables (Table I and III). However, these reliability measures during the one-leg CMJ were inferior overall compared to the two-leg CMJ. Greater variability in jump movement patterns during the one-leg CMJ was noted previously^37^ and may negate the interlimb compensation strategies used during two-leg CMJ,^37,38^ resulting in poorer intersession reliability. In particular, we found the reliability of the ND leg to be less than that of the dominant leg, suggesting that there was a degree of lower limb asymmetry present within this military cohort. Such asymmetry may indicate a potentially higher risk of MSKI, secondary to unequal force absorption and excessive loading on the stronger leg.^37,38^ While we have identified inferior reliability for the one-leg CMJ compared to the two-leg CMJ, both assessments may provide unique attributes of lower limb movement patterns during regular monitoring of physical performance and MSKI risk in the military.^37,38^

Previous studies have focused on CMJ assessments as part of their MSKI risk monitoring^17–19,22,25,26^ with less examination of balance and core strength, important contributors to MSKI risk.^2^ In the current study, the balance and plank movements were examined with the Sparta Science scores exhibiting poor-to-moderate reliability. Furthermore, the Sparta Science scores were moderately (1-8 points) lower than the Sparta Science reference ranges for the one-leg balance and one-arm plank assessments for 25- to 29-year-old adults.^39^ These lower results exemplified soldiers’ poorer balance and core strength compared to other data sets within the Sparta Science database.^39^ Furthermore, these poorer results may indicate increased variation in movement patterns (i.e., reduced stability) that contributes to the lower intersession reliability observed in the current study.^38^ Improvements in these fitness domains have been shown to be of benefit to the military with reductions in lower extremity overuse injuries obtained from military-specific training of balance (e.g., marching over uneven terrain) and core strength (e.g., heavy load carriage).^2^ Therefore, enhancement of balance and core strength may result in greater task completion and reliability of force plate metrics for future monitoring of MSKI risk.^2^

A novel component of the current study was the examination of proprietary force plate metrics. Biomechanical variables were also examined for completeness as part of the Sparta Science suite of metrics with these exhibiting similar reliability to previous work in athletes.^22–25^ Nibali et al.^22^ reported the absolute reliability (CV) of the AERC, ACF, and CVI during two-leg CMJ in elite athletes to be 21.3%, 2.7%, and 2.7%, respectively,^22^ comparable to that of the current soldier population (18.85%, 3.1%, and 3.9%, respectively, Table II). Despite this similarity, the reliability of the biomechanical variables was worse compared with the Sparta Science variables and supported earlier works.^22–24^ The poorest reliability was noted for AERC during the two-leg and one-leg CMJ and sway velocity for the one-leg balance and one-arm plank assessments (Table II and IV). Potentially, the biomechanical variables may be more sensitive to subtle changes in movement patterns, which are obscured in the proprietary algorithms of emerging technology.^16,18,19^ As such, we recommend that practitioners collect and interpret both the Sparta Science and biomechanical variables to ensure comprehensive monitoring of performance and MSKI risk using the Sparta Science system.

To our knowledge, this is the first study to examine the level of agreement for all variables documented by the Sparta Science system in a military population. Although the bias for most of the Sparta Science and biomechanical variables was small and acceptable for the cohort, the LOAs for most variables were large and unacceptable. The large LOAs indicated that sessional differences within participants were highly inconsistent between participants, highlighting the large variability of these metrics in the current cohort.^35^ Subsequently, large changes beyond the LOAs would be needed to demonstrate an improvement/deterioration of the metric in a diverse military cohort.^35^ Future studies may clarify if smaller LOAs exist for more homogenous subpopulations within the military and the practical use of these in monitoring performance and MSKI risk.

The current study makes an important and novel contribution to our understanding of the reliability of an emerging force platform system for a military population.^22–24,26^ This study engaged a substantially greater sample size than previous studies of reliability using the Sparta Science force platforms.^24,26^ Second, this study examined a range of Sparta Science assessments concurrently to assist practitioners with comprehensive monitoring of performance and MSKI risk in military personnel. Despite these unique strengths, several limitations existed. First, because of security constraints, the current study employed a prior Sparta Science software version that did not employ machine learning and a military-specific population database. Second, participants undertook concurrent military training throughout the duration of the testing period as part of their normal duties. Participants’ DOMS score at the beginning of each testing session was low with only small trivial differences between sessions and likely to be of minimal impact given that daily physical training exists for all personnel. Third, participants in this study displayed a high degree of heterogeneity in terms of age, sex, fitness levels, and occupation. Nonetheless, the current study examined participants reflective of the diverse nature of a combat brigade, mimicking the potential real-world application of an emerging technology within the military.

## CONCLUSION

The current study identified that the reliability of most of the Sparta Science and biomechanical variables during two-leg and one-leg CMJ, one-leg balance, and one-arm plank assessments was moderate to good for a military population. Specifically, greater absolute reliability was evident for the Sparta Science compared to the biomechanical variables, whereas relative reliability measures were poor to moderate, and large LOAs existed for most variables. The current findings documented the typical inconsistency in Sparta Science assessments and metrics, including minimum detectable change for meaningful changes, to assist practitioners’ use of emerging technology to monitor MSKI risk factors and/or training interventions in military personnel.

## Supplementary Material

usac387_Supp

## Data Availability

The data underlying this article will be shared on reasonable request to the corresponding author.

## INSTITUTIONAL CLEARANCE

Institutional clearance approved.
