# Supplementary figures and images for: Reliability of Force Plate Metrics During Standard Jump, Balance, and Plank Assessments in Military Personnel
Source: Mil Med. 2022 Dec 15;188(7-8):e2058–66. doi: 10.1093/milmed/usac387 (PMC10363007; doi:10.1093/milmed/usac387)

**Figure 1:** Two-leg countermovement jump test position.

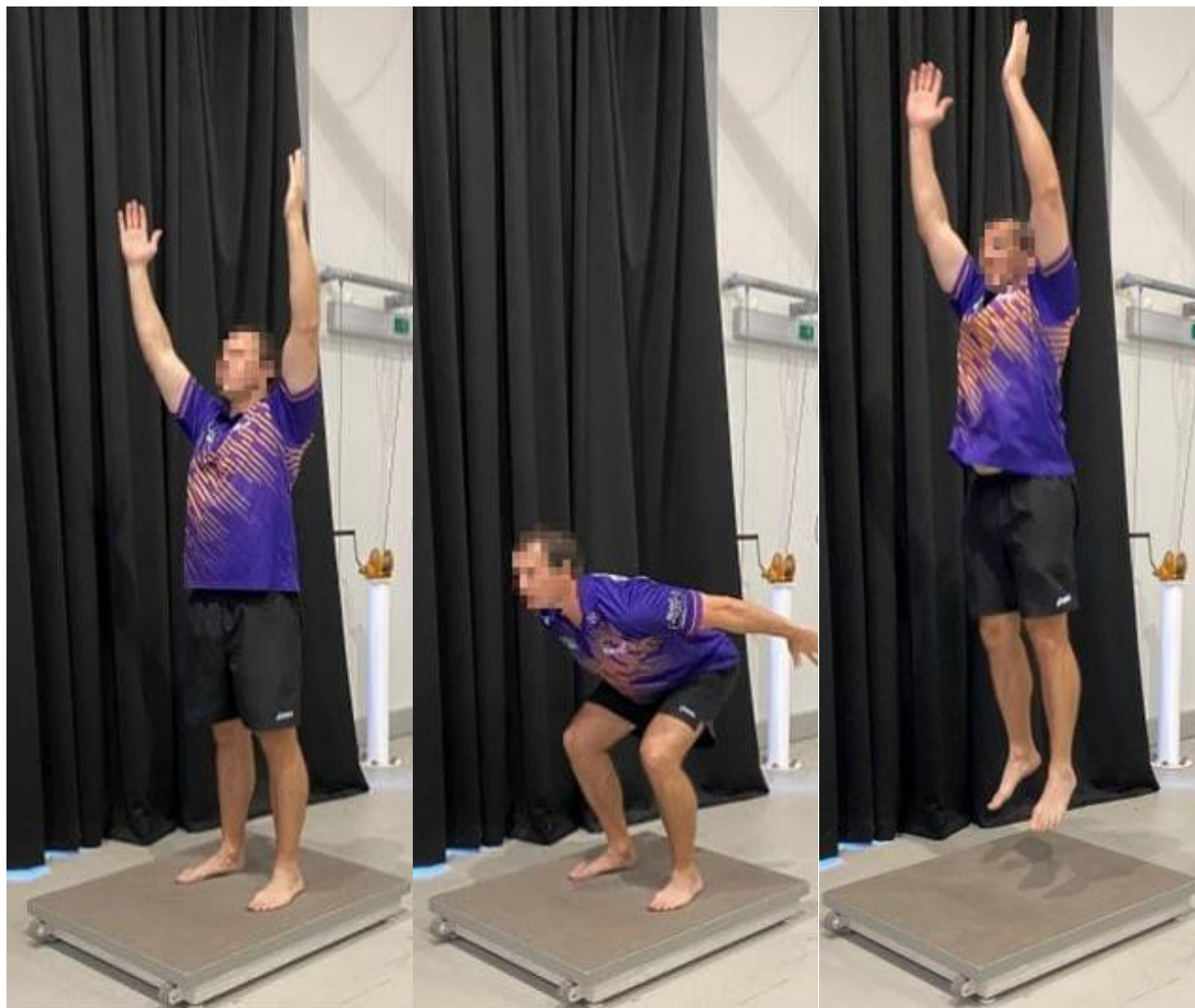

Supplement: usac387_Supp [file usac387_supp.zip › Figure 1 Two-leg CMJ.pdf]

**Figure 2:** One-leg countermovement jump test position.

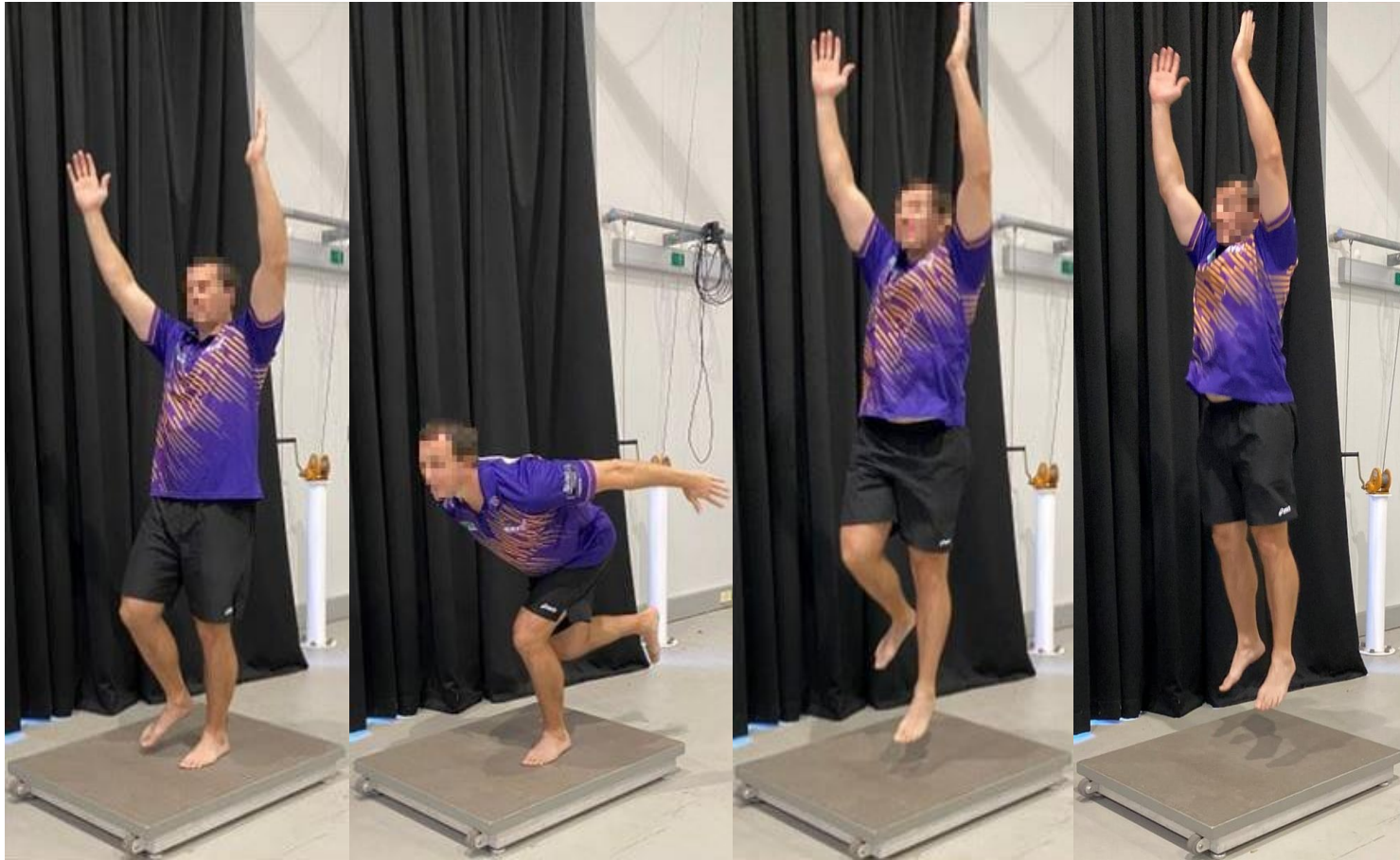

Supplement: usac387_Supp [file usac387_supp.zip › Figure 2 One-leg CMJ.pdf]

**Figure 3:** One-leg balance test position.

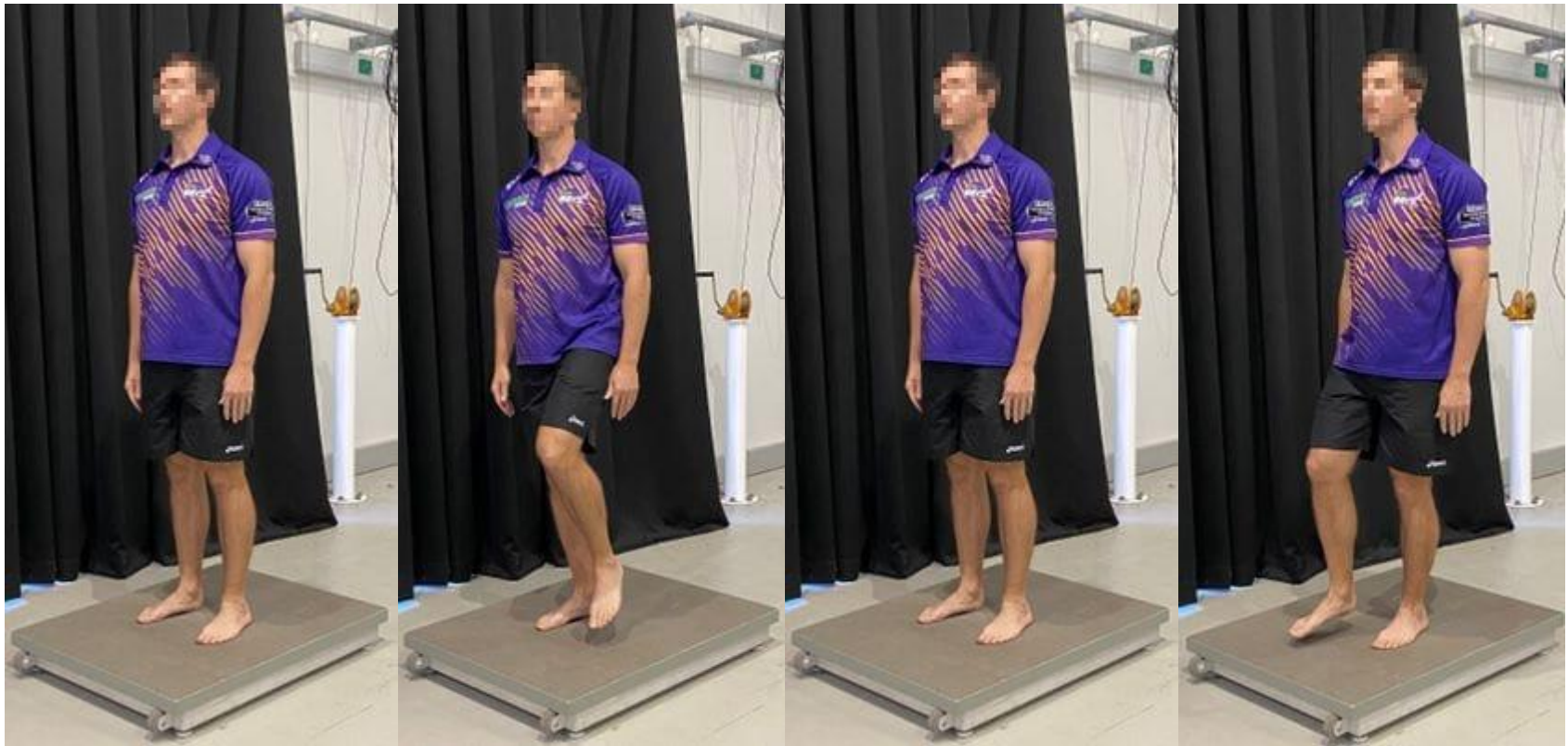

Supplement: usac387_Supp [file usac387_supp.zip › Figure 3 One-leg balance.pdf]

**Figure 4:** One-arm plank test position.

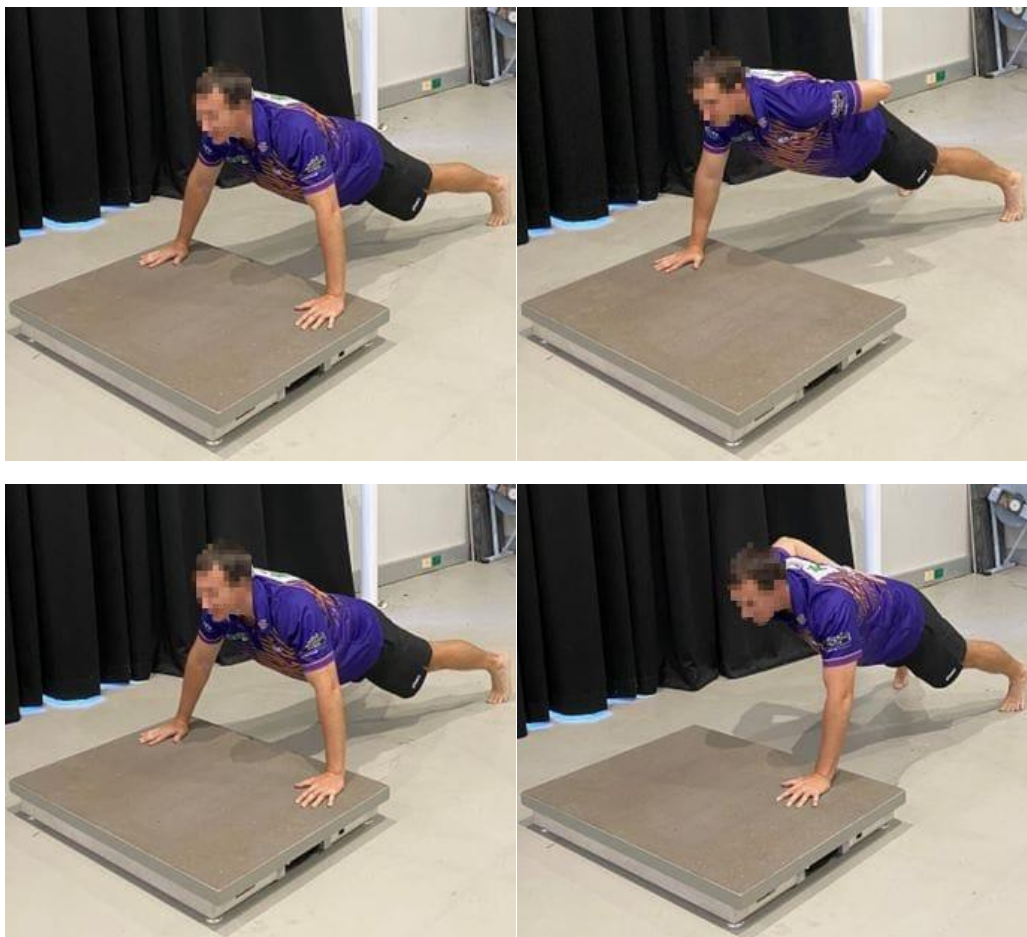

Supplement: usac387_Supp [file usac387_supp.zip › Figure 4 One-arm plank.pdf]
